# Effect of flurbiprofen axetil combined with “Cocktail” therapy on opioid dosage in patients after total knee arthroplasty

**DOI:** 10.12669/pjms.38.3.4735

**Published:** 2022

**Authors:** Lu Wang, Li-xin Wu, Zhe Han, Wen-hai Ma, Zhi-hui Geng

**Affiliations:** 1 Lu Wang, Department of Pharmacy, Baoding No.1 Central Hospital, Baoding 071000, Hebei, China; 2 Li-xin Wu, Department of Pharmacy, Baoding No.1 Central Hospital, Baoding 071000, Hebei, China; 3 Zhe Han, Third Department of Orthopedics, Baoding No.1 Central Hospital, Baoding 071000, Hebei, China; 4 Wen-hai Ma, Third Department of Orthopedics, Baoding No.1 Central Hospital, Baoding 071000, Hebei, China; 5 Zhi-hui Geng, Department of Pharmacy, Baoding No.1 Central Hospital, Baoding 071000, Hebei, China

**Keywords:** “Cocktail”, therapy, Flurbiprofen axetil, Opioids, Total knee arthroplasty

## Abstract

**Objectives:**

To investigate the effect of flurbiprofen axetil combined with “cocktail” therapy on opioid dosage in patients after total knee arthroplasty (TKA).

**Methods:**

The clinical data of 200 patients who underwent TKA in Baoding No.1 Central Hospital hospital from March 2019 to March 2021 were collected for retrospective analysis. All 200 patients were divided into two groups according to their intraoperative anesthesia methods: the control group (100 cases) and the experimental group (100 cases). Patients in the control group were treated with “cocktail” therapy intraoperatively, while those in the experimental group were treated with flurbiprofen axetil combined with “cocktail” therapy intraoperatively. The hip pain scores in resting state and motion state were compared between the two groups at different postoperative time points, and postoperative pain relief, adverse reactions, and patient satisfaction with analgesia were statistically analyzed to evaluate the postoperative quality of life of the patients.

**Results:**

A statistically significant difference was observed in the intergroup and temporal effects of pain scores in resting state and motion state between the two groups (p<0.05). By comparison at each time point, the pain scores in the experimental group were significantly lower than those in the control group at the time point T1-T6 in resting and motion states, with a statistically significant difference (p<0.05). The frequency and dosage of remedial medication per capita in the experimental group were significantly lower than those in the control group, with a statistical significance (p<0.05). There was no significant difference in the scores of life quality items between the two groups preoperatively (p>0.05), while the scores of each item in the experimental group were significantly higher than those in the control group postoperatively (p<0.05). The satisfaction degree of the experimental group was significantly higher than that of the control group, showing a statistically significant difference (p<0.05).

**Conclusions:**

Flurbiprofen axetil combined with “cocktail” therapy is a safe treatment regimen that can improve the quality of life and safety of patients. With such a regimen, postoperative pain of patients undergoing TKA can be effectively relieved, and the use of opioids can be reduced.

## INTRODUCTION

 Total knee arthroplasty (TKA), in orthopedic surgery, is a kind of surgery with large injury to the affected limb, which is characterized by relatively complicated surgery process, heavy bleeding, and severe postoperative pain. It has been shown in previous studies that the postoperative pain caused by TKA ranks the most severe among all surgical procedures, and nearly 1/5 of patients are not satisfied with the postoperative analgesic effect.^1^ Currently, there is no unified clinical standard for postoperative analgesia of TKA. “Cocktail” therapy, namely nerve block and local injection of drugs around the joint, is a commonly studied postoperative analgesia method to date.^2^ Specifically, this therapy blocks pain transmission by applying drugs directly around the nerve. Nonetheless, shortcomings are inevitable: the short duration of the drug makes it difficult to achieve the purpose of long-term analgesia, and the pain may exacerbate after the failure of analgesic drugs.^3^ Opioids are often used for remedial analgesia postoperatively, but with more serious adverse reactions. In view of this, some researchers have proposed to combine “cocktail” therapy with other analgesic methods to prolong the analgesic effect.^4^ Flurbiprofen axetil, as a non-steroidal targeted analgesic drug, mainly inhibits the generation of coxidase via spinal cord and peripheral pathways and reduces prostaglandin synthesis, thereby increasing pain threshold in the body.^5^ Its advantage over other analgesics lies in its faster action effect, relatively long action time and targeted therapy.

 Currently, few domestic studies on the combination of flurbiprofen axetil and “cocktail” therapy for analgesia have been reported. In this study, the safety and effectiveness of flurbiprofen axetil combined with “cocktail” therapy after TKA were investigated by indicators such as pain score, postoperative pain relief, and adverse reactions.

## METHODS

 The clinical data of 200 patients who underwent TKA in Baoding No.1 Central Hospital from March 2019 to March 2021 were collected for retrospective analysis.

### Inclusion criteria:

- All patients receiving TKA treatment in our hospital due to knee osteoarthritis;
- Patients classified as grade II-III according to the American Society of Anesthesiologists (ASA);
- Patients with no history of using narcotic analgesics one week before going to bed;
- Patients without previous knee surgery;
- Patients with complete clinical data.

### Ethical approval

The study was approved by the Institutional Ethics Committee of Baoding No.1 Central Hospital, (Date December 24, 2018) and written informed consent was obtained from all participants.

### Exclusion criteria:

- Patients with mental disorders or language barriers;
- Patients with allergy or tolerance to the drug in this study;
- Patients with alcohol or drug dependence;
- Patients with a long history of opioid use;
- Patients with coagulation dysfunction, severe organ lesions and short term intolerance to surgery; - Patients with severe preoperative stiffness and deformation of the knee joint or severe instability caused by collateral ligament injury.

All patients were divided into two groups according to their intraoperative anesthesia methods: the control group (100 cases) and the experimental group (100 cases). There was no significant difference in general data between the two groups, which were comparable. Table-I.

**Table I:** Comparison of general data between the two groups.

General information		Experimental group	Control group	t/χ^2^	p
Gender (cases)	Male	23	26	0.243	0.622
Age (years old)	Female	77	74		
Body mass index (kg/m^2^)		66.35±5.52	66.22±5.93	0.164	0.870
Weight (kg)		27.18±3.84	27.62±3.92	0.802	0.424
		69.38±11.47	67.27±11.43	0.952	0.343
ASA grading (cases)	Grade II	62	67	0.546	0.460
	Grade II	38	33		
Affected knee position (cases)	Left knee	54	52	0.080	0.777
	Right knee	46	48		

### Methods

Control group: The cocktail was prepared into a 100 ml solution consisting of 200 mg of ropivacaine injection (Astra Zeneca AB, Import Drug Registration Certificate H20140763) + 5mg of morphine hydrochloride injection (Northeast Pharmaceutical Group Company Shenyang No.1 Pharmaceutical Co., Ltd., National Drug Approval No. H21022436) + normal saline. 50ml of the above cocktails were extracted and added into 40mg of triamcinolone acetonide injection (Kunming Jida Pharmaceutical Co., Ltd., State Drug Approval No. H53021604). 50ml of the above cocktail was drawn and added 40mg of triamcinolone acetonide injection (Kunming Jida Pharmaceutical Co., Ltd., National Medicine Standard H53021604), with the injection site at the posterior joint capsule; 50ml cocktail without hormones was injected into MCL, LCL, quadriceps, subpatellar fat pad, other joint capsules and subcutaneous locations. Experimental group: 50mg flurbiprofen axetil injection (Beijing Tide Pharmaceutical Co., Ltd., State Drug Approval No. H20041508) was added into the above cocktails, and the other drug use and administration methods were the same as those in the control group. Remedial analgesia regimen: Patients were given dezocine (Yangtze River Pharmaceutical Group Co., Ltd., State Drug Approval No. H20080329) intramuscular injection in case of pain visual analogue scale (VAS) ≥ 4 points.

### Observation indicators:

### Pain score

VAS was used to evaluate the pain degree of patients in both groups in the resting state and motion state at 6 time points of 6 h (T1), 12 h (T2), 24 h (T3), 48 h (T4), 72 h (T5) and 5 d (T6) postoperatively. 0 points indicates painless and 10 points means intolerable severe pain. The higher the score, the more severe the pain.

### Postoperative pain relief

The number of postoperative pain relief and the dosage of remedial drugs per capita in the two groups were statistically analyzed.

### Analgesia-related adverse reactions

The incidence of postoperative adverse analgesic reactions, such as malignant vomiting and respiratory depression, was counted in the two groups.

### Postoperative quality of life

The World Health Organization Quality of Life Scale (WHOQOL-BREF)^6^ was used to evaluate preoperative and postoperative quality of life, including the fields of physiology, psychology, social relations and environment. The postoperative quality of life of each patient was scored using a 1-5 scale, which was converted to a percentage scale. A higher score indicates a better quality of life.

### Satisfaction

Patients’ satisfaction was recorded during analgesia, and the satisfaction was divided into very satisfied (I), satisfied (II), fair (III) and poor (IV).

### Statistical Analysis

SPSS20.0 statistical software was used to analyze the data. Enumeration data were presented as N (%), χ2 or continuum-corrected χ2 test was performed, and rank sum test was used for grade data. Measurement data was expressed as *x*¯±*s*, and t-test was performed. Multiple time points were compared by repeated measurement variance, and further pairwise comparison was performed by LSD-t test. P<0.05 was considered statistically significant.

## RESULTS

 A statistically significant difference was observed in the inter-group and temporal effects of pain scores in resting state and motion state between the two groups (p<0.05). By comparison at each time point, the pain scores in the experimental group were significantly lower than those in the control group at the time point T1-T6 in resting and motion states, with a statistically significant difference (p<0.05). Table-II.

**Table II:** Comparison of postoperative pain scores between the two groups (*x*¯±*s*, points).

Group		T1	T2	T3	T4	T5	T6
Resting state	Experimental group	1.26±0.38	2.14±0.62	2.18±0.67	2.25±0.56	2.07±0.43	1.53±0.61
	Control group	1.38±0.41	2.34±0.59	2.65±0.63	3.06±0.60	3.18±0.51	2.43±0.75
	F_inter-group_, P_inter-group_			330.4,p<0.001			
	F_time_, P_time_			153.12,p<0.001			
	F_interaction_, P_interaction_			24.39,p<0.001			
Motion state	Experimental group	1.54±0.53	2.26±0.47	2.37±0.54	2.42±0.46	2.38±0.44	2.09±0.68
	Control group	1.85±0.61	3.09±0.71	3.49±0.58	3.78±0.59	4.05±0.56	3.28±0.49
	F_inter-group_, P_inter-group_			33.35,p<0.001			
	F_time_, P_time_			13.75,p<0.001			
	F_interaction_, P_interaction_			5.901,p<0.001			

 The frequency and dosage of remedial medication per capita in the experimental group were significantly lower than those in the control group, with a statistical significance (p<0.05). Table-III. The incidence of nausea and vomiting in the experimental group was significantly lower than that in the control group (p<0.05). No significant difference was observed in the incidence of other adverse reactions between the two groups (p>0.05). Table-IV. There was no significant difference in the scores of life quality items between the two groups preoperatively (p>0.05), while the scores of each item in the experimental group were significantly higher than those in the control group postoperatively (p<0.05). Table-V. The satisfaction degree of the experimental group was significantly higher than that of the control group, showing a statistically significant difference (p<0.05). Table-VI.

**Table III:** Comparison of postoperative pain relief between the two groups (*x*¯±s ).

Group	Number of remedial medication per capita (times)	Remediation dose per capita (mg)
Experimental group	0.93±0.29	71.54±35.70
Control group	1.07±0.45	89.55±42.09
t	2.615	3.025
p	0.010	0.003

**Table IV:** Comparison of analgesia-related adverse reactions between the two groups [n(%)].

Group	Nausea and vomiting	Inadequate analgesia	Erythra	Respiratory depression	Dizziness
Experimental group	3(3.00)	2(2.00)	2(2.00)	1(1.00)	4(4.00)
Control group	10(10.00)	6(6.00)	5(5.00)	3(3.00)	9(9.00)
χ2 or continuity correction	4.031	1.172	0.592	0.255	2.057
*p*	0.045	0.279	0.442	0.614	0.152

**Table V:** Comparison of postoperative quality of life between the two groups (*x*¯±s, points).

Item		Experimental group	Control group	t	p
Physical field	Before surgery	56.79±5.32	57.29±4.91	0.691	0.491
	After surgery	65.75±6.18	63.34±7.28	2.524	0.012
Psychological field	Before surgery	53.42±3.09	53.98±2.86	1.330	0.185
	After surgery	59.17±4.46	57.15±5.37	2.894	0.004
Social relations field	Before surgery	55.71±4.28	56.23±3.82	0.906	0.366
	After surgery	62.75±3.25	60.07±3.59	5.534	<0.001
Environmental field	Before surgery	63.26±5.81	62.93±5.43	0.415	0.679
	After surgery	70.08±4.67	68.74±3.95	2.190	0.030

**Table VI:** Comparison of patient satisfaction between the two groups [n(%)].

Group	I	II	III	IV
Experimental group	48 (48.00)	37 (37.00)	13 (13.00)	2 (2.00)
Control group	37 (37.00)	35 (35.00)	20 (20.00)	8 (8.00)
Z	4.802			
p	0.028			

## DISCUSSION

 TKA is considered to be an effective surgical procedure for the treatment of knee joint disease and osteoarthritis. However, it was found in Rice Da et al.^7^ study that 20%-70% of patients who received TKA had moderate to severe pain during the first 1-4 day. Failure to adequately control pain will not only lead to pathophysiological reactions, increase patient negative emotions, disrupt sleep, and reduce patient satisfaction postoperatively, but also increase the risk of complications such as deep vein thrombosis, wound infection, and delayed healing.^8^,^9^ Opioids, as first-line analgesics, boast a preferable analgesic effect, but the effect of dose control and postoperative pain treatment is difficult to be achieved due to its serious side effects.^10^ In view of this, a more effective analgesic regimen remains to be sought.

 “Cocktail” therapy was first proposed by Australian scholars, which refers to the implementation of local infiltration analgesia by injecting local anesthetics, opioids or non-steroidal drugs into the posterior capsule of the knee joint and soft tissues around the surgical area by adding adrenaline or corticosteroids and other drugs.^11^ A previous study by Danoff Jr et al.^12^ showed that “cocktail” therapy used for TKA significantly reduced the severity of early acute pain and promoted early functional exercise of the knee joint. Moreover, a study by Chai X et al.^13^ discussed many cases and also found that “cocktail” therapy can produce a preferable analgesic effect, with no significant increase in the incidence of adverse reactions. Flurbiprofen axetil is one of the clinically commonly used non-steroidal anti-inflammatory drugs (NSAIDs) in major orthopedic surgery. It has a high affinity for inflamed tissues and can achieve targeted drug therapy and prolong the action time.^14^ However, due to the limited analgesic effect of NSAIDs, flurbiprofen axetil is only an effective auxiliary analgesic of opioids for moderate to severe pain. It was shown in a study by Xiao X et al.^15^ that flurbiprofen axetil combined with opiates can have a stronger analgesic effect in patients with TKA. In this study, flurbiprofen axetil combined with “cocktail” therapy is used in the postoperative analgesia of patients with TKA. The results show that a statistically significant difference can be observed in the inter-group and temporal effects of pain scores in resting state and motion state between the two groups. By comparison at each time point, the pain scores in the experimental group are significantly lower than those in the control group at the time point T1-T6 in resting and motion states, with a statistically significant difference, and the frequency and dosage of remedial medication per capita in the experimental group are significantly lower than those in the control group, suggesting that the combination of the two analgesic regimens can more effectively relieve the acute pain after TKA and reduce the use of opioids compared to “cocktail” therapy alone. The reason may have a close bearing on the inhibition of inflammatory response at the surgical site and the alleviation of inflammatory pain. TKA, during the intraoperative surface repair of the knee joint, usually requires a large number of bone removal and release of surrounding soft tissues, which causes great trauma and stress response to the body, resulting in severe postoperative pain. Meanwhile, tourniquets need to be used intraoperatively to reduce bleeding and expand surgical field of vision, which will stimulate the body to release inflammatory factors and increase the degree of postoperative pain.^16^,^17^ In “cocktail” therapy, ropivacaine can inhibit sodium channels on nerve cells and block nerve excitatory conduction; As opioids, morphine can bind to the central and surrounding opioid receptors, inhibit the pain-sensing area of the cerebral cortex, and produce strong analgesic effect. Triamcinolone acetonide is conducive to alleviating the stress response caused by local trauma and also relieving postoperative pain.^18^,^19^ Under the combined action of the above three drugs, the stimulation of the sensory nerve endings by the pain factors produced by local tissue damage can be blocked, thereby reducing the transmission of pain from the nerve endings to the central nervous system and achieving the purpose of controlling postoperative pain. In contrast, flurbiprofen axetil can inhibit cyclooxygenase to reduce arachidonic acid to produce prostaglandins, and at the same time inhibit the production of central prostaglandins, thereby reducing the sensitization of the central and peripheral nervous systems and achieving analgesic effects.^20^,^21^ It was shown in a study by Wang J et al.^22^ that flurbiprofen axetil can be targeted to accumulate on surgical incisions and inflammation sites, with rapid onset, and the blood concentration can reach the peak 5-10 minutes after intravenous administration. With the combination of two analgesic regimens, the analgesic effect can be synergistically exerted and the analgesic effect can be optimized.

 Furthermore, the incidence of nausea and vomiting in the experimental group is significantly lower than that in the control group, and the quality of life score and satisfaction are significantly higher than those in the control group, suggesting that flurbiprofen axetil combined with “cocktail” therapy is safer and can improve the postoperative quality of life and satisfaction degree of analgesia in patients. To explain it, flurbiprofen axetil does not produce central side effects similar to morphine respiratory depression. It was confirmed by Wang X et al.^23^ that flurbiprofen axetil can not only reduce inflammatory reaction and induce neuroprotective effect, but also overcome the adverse effects of other COX inhibitors and reduce the occurrence of gastric mucosal adverse reactions.^24^ In addition, both flurbiprofen axil and “cocktail” therapy have been proved to have neither significant effect on the motor nerve of patients nor adverse effect on postoperative recovery of knee function in patients with TKA. With the improvement of postoperative analgesia, not only can the satisfaction of patients be improved, but also their quality of life can be improved by promoting early postoperative functional exercise.^25^

### Limitations of the study

Nevertheless, deficiencies are still visible in this study: VAS is used to score the pain, and the results are subjective to a certain extent due to the different tolerance and expression of pain in different patients. At the same time, the short postoperative observation and follow-up time leads to the lack of comparison with long-term effects. In view of this, more rigorous experimental methods are expected to be designed in future studies to improve the above deficiencies.

## CONCLUSIONS

Flurbiprofen axetil combined with “cocktail” therapy is a safe treatment regimen that can improve the quality of life and safety of patients. With such a regimen, postoperative pain of patients undergoing TKA can be effectively relieved, and the use of opioids can be reduced.

### Authors’ Contributions

**LW** & **LXW:** Designed this study and prepared this manuscript,and are responsible and accountable for the accuracy or integrity of the work. **ZHhG:** Collected and analyzed clinical data. **ZH & WHM:** Were involved in the study and significantly revised this manuscript.
